# Which On-Pack Information Drives a Marketable Specialty Coffee Label? Unfolding Purchase Intention and Visual Attention with Eye Tracking

**DOI:** 10.3390/foods14244235

**Published:** 2025-12-09

**Authors:** Alexandre H. Silas Souza, Louise P. Passos, Katiúcia Alves Amorim, Maria Galdino, Jéssica Sousa Guimarães, André Pimenta Freire, Cleiton Antonio Nunes, Ana Carla Marques Pinheiro

**Affiliations:** 1 Department of Food Science, Federal University of Lavras, DCA/UFLA, Lavras 37200-900, Brazil; paiva.louise@gmail.com (L.P.P.); katiucianutri@gmail.com (K.A.A.); maria.galdino@estudante.ufla.br (M.G.); jsguimaraes.nutri@gmail.com (J.S.G.);; 2 Department of Computer Science, Federal University of Lavras, DCC/UFLA, Lavras 37200-900, Brazil; apfreire@ufla.br

**Keywords:** sensory analysis, consumer behavior, biometric sensor, packaging, landscape segmentation analysis

## Abstract

This study examined how visual attention to specialty coffee label elements relates to consumers’ stated purchase intention. A total of 105 regular specialty coffee consumers viewed the front and back panels, simultaneously, of six commercially available labels while their eye movements were recorded with an eye tracker. Areas of Interest (AOIs) were defined for the label’s content, and a Normalized Fixation Ratio (NFR; proportional fixation time scaled by AOI area) was calculated. Purchase intention was measured on a seven-point structured scale, and the association between NFR and purchase intention was modeled using Landscape Segmentation Analysis (LSA). Heatmaps showed that central regions of the front and back panels were attentional “hot zones”, particularly when they contained sensory claims, cupping score, origin and traceability, roast level, coffee variety, and the “specialty coffee” designation. In contrast, weight, best-before date, grain or ground, and contact information consistently received little attention. Higher NFR values for sensory and origin-related cues were positively associated with purchase intention; labels that gave these attributes visual prominence achieved the highest intention scores. These findings indicate that consumers prioritize sensory and traceability-related information over technical or administrative cues and that both the content and graphic salience of label elements are critical for driving perceived value and choice. Results provide evidence-based guidance for structuring specialty coffee labels to optimize communication.

## 1. Introduction

Coffee is deeply embedded in global culture, and its consumption has evolved alongside broader socio-economic transformations. Since the Industrial Revolution, coffee has been linked to heightened concentration and cognitive performance, helping to consolidate its worldwide popularity and everyday relevance [1,2]. Over the last decade, international markets have witnessed sustained growth in specialty coffee, driven by rising consumer interest in quality differentiation, provenance, sustainability, and distinctive sensorial profiles. Global outlooks and market analyses document these shifts in preferences and value perception, underscoring a broader premiumization of food and beverage categories [3,4,5,6,7].

Within this global landscape, labeling has become a critical interface in producer–consumer communication. Labels convey complex combinations of intrinsic and credence attributes (e.g., origin, processing, roast level, certifications of sustainability or authenticity, and sensory claims)that shape expectations, perceived quality, and ultimately purchasing decisions [6,7,8,9]. As a result, label design and information architecture are increasingly treated as strategic tools for market positioning and consumer education across food systems. This role is particularly pronounced for specialty coffee, where products are often sold as beans or ground coffee to be brewed later, so that the label becomes the primary source of information used to justify price premiums and choose between origins, sensory claims, and roasting styles.

To understand how consumers actually interact with on-pack information, eye tracking has emerged as a powerful method to capture objective metrics of visual attention (e.g., fixation duration and distribution) on specific label elements [10]. Its application in sensory and consumer science spans the salience of health/nutrition cues [11] and comparative evaluations of label configurations in diverse product categories such as yogurts and chocolates [12,13]. Recent studies have explicitly linked visual attention to choice outcomes in food labeling: Van Loo et al. [6] showed that increased fixation on nutrition and sustainability claims was associated with a higher probability of product choice; Peschel et al. [7] reported that enlarging and visually enhancing logos and other design elements increased both attention and selection likelihood; and Zamani et al. [14] found that the number of fixations on specific packaging areas (e.g., colors, images and size cues) can predict consumer preferences. These studies demonstrate that what consumers look at can diverge from what they say they value, highlighting the importance of combining implicit and explicit measures.

Despite these advances, important gaps remain for specialty coffee labels. Eye tracking research (passive methods) by Teixeira et al. [15] indicates strong attention to sustainability seals and roast, but comparatively weaker attention to altitude and variety; conversely, active-method studies [1,16] report that consumers explicitly value those very attributes, revealing a potential discrepancy between stated importance and actual on-label visual engagement. More broadly, passive methods capture involuntary, moment-to-moment allocation of attention [17,18], whereas active methods, such as questionnaires and rating scales, elicit conscious judgments and articulated preferences [19]. Integrating both perspectives is, therefore, essential for a more complete account of how label information informs expectation formation, perceived quality, and choice.

Accordingly, this study integrates eye tracking (passive) with explicit purchase intention (active) to examine how visual attention to specialty coffee label elements relates to consumers’ stated propensity to buy. Using six real labels currently available in the marketplace and a sample of 105 regular specialty coffee consumers, we analyze fixation duration across Areas of Interest (AOIs) to identify which types of information exert the greatest influence on purchase intention. In doing so, the study directly addresses the current gap regarding how consumers navigate the highly information-rich environment of specialty coffee packaging. By linking attention patterns to behavioral intent, our goal is to generate evidence-based guidance for more effective label design that optimizes producer—consumer communication and adds value to the global specialty coffee market [20,21,22].

## 2. Materials and Methods

### 2.1. Experiment Overview

The study was approved by the Research Ethics Committee of the Federal University of Lavras (CAAE: 76394223.0.0000.5148), and all participants provided written informed consent before testing. A total of 105 regular specialty coffee consumers (18–50 years; 69% women; 79.8% with higher education) took part. A screening questionnaire characterized consumption habits: 85% reported consuming specialty coffee more than once per week. Regarding typical points of purchase, 63% bought from coffee shops, 57% directly from producers, 47% from supermarkets, 38% from gourmet stores, 31% via online platforms, and 3% from neighborhood markets.

The experiment was conducted in a controlled, private room at the Sensory Analysis Laboratory (Department of Food Science, UFLA), with standardized temperature, lighting, and noise to promote evaluator focus. Participants viewed six digitized coffee labels while their visual behavior was tracked using a Tobii Pro Fusion (120 Hz), which recorded gaze-fixation duration. After viewing, participants indicated purchase intention on a seven-point structured scale.

### 2.2. Samples

Labels were collected from retail outlets in Lavras, Minas Gerais (Brazil), and during the International Coffee Week (SIC), an annual event that gathers ~17,000 stakeholders across the coffee chain (producers, buyers, exporters, researchers, baristas, and consumers). In total, 31 specialty coffee labels commercially available in the Brazilian market were obtained.

Labels were first screened according to Brazil’s Portaria SDA No. 570 (9 May 2022), which specifies mandatory coffee-label information. Labels missing any required elements—roast level, coffee species (Arabica, Robusta, or blend), or product form (ground or whole bean)—were excluded. After this compliance filter, 10 labels remained.

To reduce redundancy and ensure informational diversity in the final set, a binary matrix was constructed linking each label to the information elements present on its packaging, enabling comparative analysis. A Correspondence Analysis (XLSTAT 2025.1) was then applied to map similarities and differences among labels. The analysis included 23 informational elements (e.g., organic claim, women’s movement, sensory claim, Specialty Coffee Assosciation (SCA) score, variety, roast level, best-before date, QR code, and contact information). Using the Correspondence Analysis map, six labels were selected as representative of the informational diversity observed in the initial sample.

### 2.3. Eye Tracking Assessment

Visual behavior toward specialty coffee labels was measured using eye tracking, enabling precise quantification of attentional allocation during label interaction. Data were captured with a Tobii Pro Fusion 120 Hz (Stockholm, Sweden) eye tracker coupled to a 24-inch full HD LED monitor displaying digitized, bidimensional label stimuli in full screen, with the front panel on the left and the back panel on the right simultaneously. This configuration resulted in an on-screen representation of approximately 35 cm in height and 25 cm in width for the front panel and 25 cm in width for the back panel, closely matching the physical dimensions of 250 g specialty coffee packages commonly found at retail. Tobii Pro Lab (v1.98) software was used for device calibration, gaze recording, data extraction, and heatmap generation.

Participants were seated at a comfortable viewing distance and position, typically around 60–70 cm from the screen; the chair and monitor height were adjusted individually so that, although the exact visual angle varied slightly between participants, it remained within the recommended operating range of the eye tracker and was verified during calibration.

Prior to testing, an individual calibration procedure was performed in Tobii Pro Lab, as recommended by the manufacturer, to ensure data accuracy. The calibration procedure was repeated whenever the calibration plot indicated poor fit or unstable gaze estimation, until an acceptable tracking level was obtained following the manufacturer’s guidelines. If a satisfactory calibration could not be achieved after repeated attempts, the participant was thanked and dismissed, and no eye tracking data were collected for that session.

During the task, each participant viewed the six selected labels and freely explored each one for as long as they wished, with no minimum or maximum viewing time imposed; participants manually advanced to the next label when they decided to move on, simulating a natural product-choice scenario, while fixation duration was continuously recorded. The version of the software used did not allow automatic randomization or full counterbalancing of label order. To mitigate potential order and fatigue effects associated with a strictly fixed sequence, the presentation order of the six labels was manually rearranged in a randomly chosen sequence after every five participants, so that different subgroups of consumers were exposed to different label orders.

Eye tracking data quality was inspected for each participant after recording, and datasets with a tracking ratio below 80% were excluded from the analysis; all included datasets (*n* = 105) had a tracking ratio equal to or above this threshold.

Following data collection, gaze records were processed in Tobii Pro Lab with segmentation into Areas of Interest (AOIs) to extract metrics for specific label elements. AOIs were defined as spatial regions on the label that could include both textual and graphical elements related to a given concept, rather than a single type of object (e.g., only text or only logos). For example, the “sustainability” AOI aggregated both seals and short textual claims related to environmental certifications or eco-responsible practices. The AOI set included social-appeal marketing, traceability, grain or ground, coffee species, weight, roast level, best-before date, preparation method, contact information, specialty/gourmet, SCA score, sensory claims, coffee variety, post-harvest processing, and sustainability.

### 2.4. Purchase Intention Assessment

Purchase intention was measured to gauge consumers’ predisposition to buy the specialty coffees depicted on the evaluated labels. A horizontal, 7-point, verbal numeric, bipolar scale (widely used in consumer research)was employed. Participants indicated their likelihood of purchase using the categories “Certainly would not buy,” “Probably would not buy,” “Might not buy,” “Not sure if I would buy,” “Might buy,” “Probably would buy,” and “Certainly would buy,” coded from 1 to 7, respectively, yielding a quantitative purchase-intention score [22]. Data collection occurred immediately after the eye tracking task to ensure responses reflected the visual processing of the labels with minimal external interference. Individual responses were recorded in Compusense software (v25.0.4), ensuring standardized administration and data integrity. This global judgment captured consumers’ overall disposition to purchase, considering all visual stimuli present on each label.

### 2.5. Coffee Consumption Behavior Questionnaire

Following the eye tracking and purchase-intention phases, participants completed a structured questionnaire to characterize coffee consumption habits. Administered in Compusense, the survey ensured consistent data collection. Items covered gender, age group, frequency of coffee consumption, and primary points of purchase (e.g., beans or ground coffee from coffee shops, directly from producers, supermarkets, specialty stores, or online platforms). These measures enabled the assessment of participants’ engagement with specialty coffee and provided contextual variables for interpreting attentional and purchase-intention outcomes.

### 2.6. Data Analysis

For the eye tracking analysis, Areas of Interest (AOIs) on the coffee labels were predefined in Tobii Pro Lab (Tobii Technology, Stockholm, Sweden). As emphasized by [23], AOI creation is essential to delimit specific parts of a stimulus—such as a word in a sentence or an object in a scene—thereby enabling the measurement of how long and how often viewers direct their gaze to those regions. AOIs were defined to demarcate label zones devoted to specific attributes (e.g., roast level, ground vs. whole bean, sensory claims, mandatory information, and the cupping score reported under the Specialty Coffee Association protocol). This AOI scheme enabled the extraction of targeted visual-attention metrics, particularly total fixation time per AOI. Fixation time corresponds to the accumulated duration (in milliseconds) for which a participant’s gaze remained within a given AOI [20].

To ensure comparability across participants and labels, fixation data were normalized individually. First, the proportional fixation time for each AOI was calculated by dividing the fixation time on the AOI by the total fixation time on the respective label, using the following equation:

$$T_{\mathrm{prop}}=\frac{T_{\mathrm{fix}\_\mathrm{AOI}}}{T_{\mathrm{total}\_\mathrm{label}}}$$

where

- $T_{\mathrm{prop}}$ = proportional fixation time;
- $T_{\mathrm{fix}\_\mathrm{AOI}}$ = fixation time on the specific AOI (in milliseconds);
- $T_{\mathrm{total}\_\mathrm{label}}$ = total fixation time on the entire label (in milliseconds).

In parallel, the relative area of each AOI was computed using the following equation:

$$A_{\mathrm{rel}}\;=\;\frac{A_{\mathrm{AOIs}}}{A_{\mathrm{total}\_\mathrm{label}}}$$

where

- $A_{\mathrm{rel}}$ = relative area of the AOI;
- $A_{\mathrm{AOIs}}$ = area of the specific AOI (in cm^2^);
- $A_{\mathrm{total}\_\mathrm{label}}$ = total label area (in cm^2^).

Based on these values, the relative fixation time was then calculated. This metric corresponds to the ratio between the proportional fixation time and the relative area of the AOI, according to the following equation:

$$T_{\mathrm{rel}}=\frac{T_{\mathrm{prop}}}{A_{\mathrm{rel}}}$$

where

T_rel_ = Normalized Fixation Ratio (NFR)

To relate visual attention to purchasing propensity, we modeled the association between AOI-level Normalized Fixation Ratios (NFRs) and purchase intention (PI) using Landscape Segmentation Analysis (LSA), implemented in IFPrograms. LSA begins by “unfolding” individual consumer PI responses into a preference space via a probabilistic similarity model, estimating consumer ideal points and generating a contour map that captures potential nonlinear relationships and localized optima in liking/choice. In this study, a two-dimensional unfolding solution was retained, explaining together 82% of the variance in PI while allowing a straightforward graphical representation of consumers’ involvement with the label’s displayed information. Descriptive predictors—in our case, NFR values per AOI—are then regressed onto the LSA surface to identify drivers of purchase intention. This approach is preferable to linear preference mapping when relationships are curved or saturating, and when multiple attributes jointly determine choice. We implemented LSA following current best-practice descriptions in sensory/consumer science and interpreted AOIs with positive local gradients toward higher PI as putative drivers [24].

## 3. Results

### 3.1. Evaluated Labels

**Figure 1:**
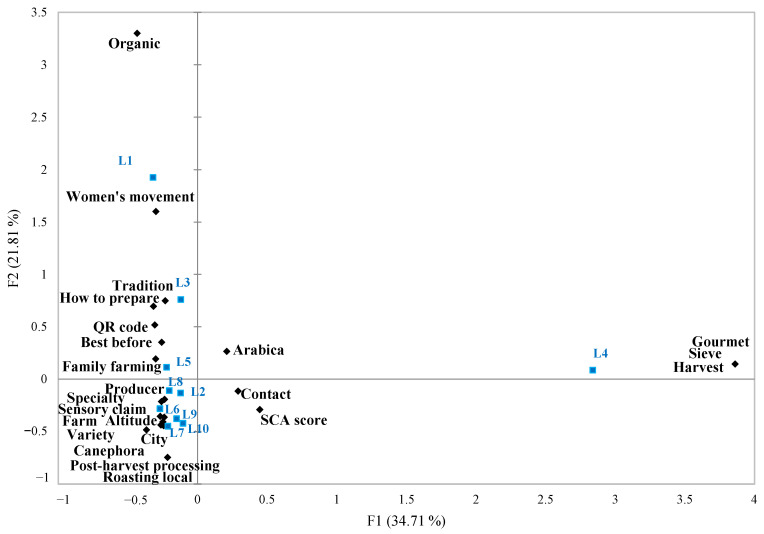
Correspondence Analysis biplot linking specialty coffee labels (blue squares) to their declared on-pack information (black diamonds).

To ensure informational diversity in the sample and reduce visual fatigue among participants, a Correspondence Analysis (CA) was performed to eliminate labels that were overly similar to one another (Figure 1).

The analysis (Figure 1) associated each coffee label with the set of on-pack information present. CA results indicated that labels L1, L3, L5, and L4 were differentiated from the remainder by containing distinct informational profiles and were, therefore, selected. In addition, labels L2 and L6 were chosen to represent the large cluster identified in the third quadrant of the CA map. The final selection comprised six labels, preserving the breadth of information observed across commercial specialty coffee packages while controlling redundancy.

### 3.2. Eye Tracking

**Figure 2:**
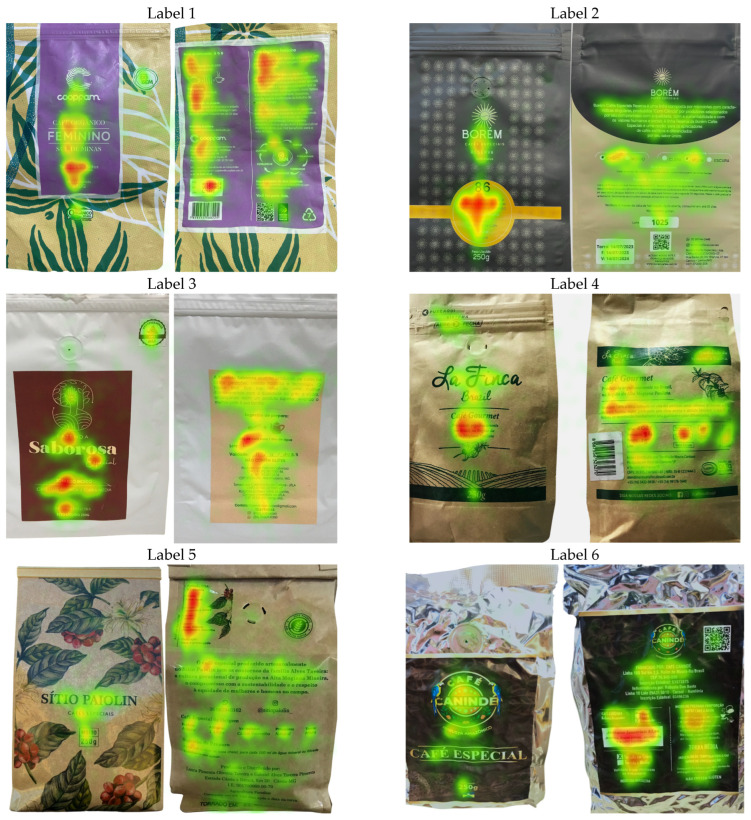
Heatmaps of consumer visual fixations on the evaluated specialty coffee labels (front and back panels). Red/orange areas signify high fixation density and green/yellow areas lower density.

To identify which label elements most strongly capture attention, heatmaps were generated (Figure 2) from fixation-duration data exported from Tobii Pro Lab. In these maps, warm colors (red/orange) denote high fixation density, whereas cool colors (green/yellow) indicate a lower concentration of gaze. This visualization clarifies which regions attract attention and how such patterns may shape perception and purchase-related judgments [25].

On the front panel, a consistent pattern emerged across labels: “hot zones” clustered in the upper-central and mid-central regions, typically occupied by the logo, brand name, or salient claims (e.g., “specialty coffee”), which concentrated most initial fixations. The lower-central area(where net weight often appears) also drew recurring, though more modest, attention, whereas lower lateral edges and (to a lesser extent) upper corners showed low fixation densities, likely because little relevant information was placed there rather than any inherent avoidance of those positions.

The back panel displayed greater variability, with fixation patterns shifting according to each label’s information architecture. For example, Label 5 concentrated attention in the upper-left quadrant containing sensory claims; Label 1 showed gaze tracking along the margins of narrative text, with longer dwells on the opening lines; Label 6 exhibited a bipartite pattern between sensory claims and icon-based preparation instructions; Label 3 split attention between preparation tips and best-before information; and Label 4 showed a relatively uniform distribution across upper and central regions, with limited engagement in the lower band.

**Figure 3:**
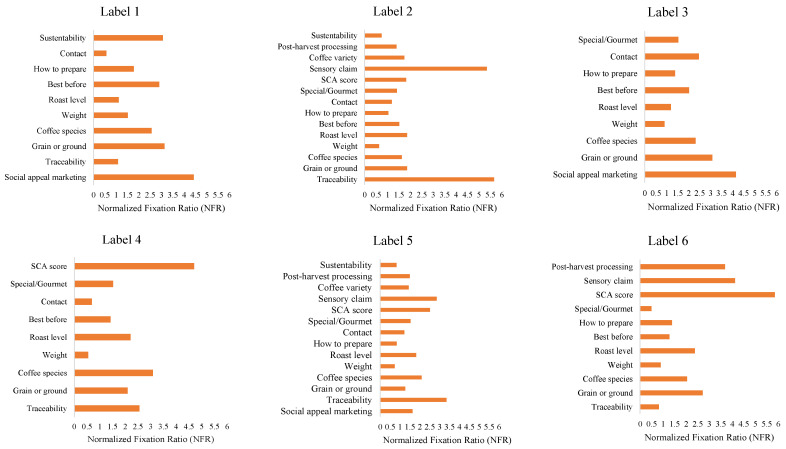
Normalized Fixation Ratio (NFR) across the analyzed coffee labels.

Figure 3 displays the distribution of the Normalized Fixation Ratio (NFR) across the analyzed coffee labels. NFR is computed as the ratio between the proportional fixation time on each AOI and its relative area on the label. This normalization enables fair comparisons among AOIs of different sizes and highlights which information truly captured visual attention beyond mere area effects.

Across the six coffee labels, attributes with narrative or evaluative appeal consistently attracted higher proportional attention than basic informational fields. On Label 1, social-appeal marketing showed the highest NFR, followed by sustainability. Intermediate values were observed for best before, grain or ground, and coffee species, whereas how to prepare, roast level, traceability, contact, and weight exhibited low NFRs.

Label 2 was dominated by traceability(the peak for this label) alongside a strong sensory claim. SCA score and best before formed an intermediate tier, while weight, sustainability, and contact remained among the least attended. On Label 3, social-appeal marketing was again the most attended attribute, followed by grain or ground; coffee species and contact were mid-range, and roast level and weight were relatively low.

Label 4 displayed a clear maximum for SCA score, with coffee species, traceability, and roast level in the middle of the distribution; contact, weight, and special/gourmet were the least attended. For Label 5, social-appeal marketing yielded the highest NFR, with sensory claim in the second tier; most other attributes—including sustainability, roast level, coffee species, weight, and traceability—clustered at low values. Finally, Label 6 was led by SCA score, followed by sensory claim and post-harvest processing. In contrast, special/gourmet, traceability, and weight were among the lowest.

A cross-label synthesis highlights three robust tendencies. First, when present, sensory claims reliably rank among the most attended elements (Labels 2, 5, and 6), underscoring the salience of direct promises about product experience. Second, the SCA score attracts high attention where it is visually prominent (Labels 4 and 6), suggesting evaluative scores are strong attentional magnets. Third, weight repeatedly appears among the least attended attributes (five of six labels), often accompanied by contact. Additionally, social-appeal marketing attains high NFR whenever present (Labels 1, 3, and 5).

### 3.3. Purchase Intention

**Figure 4:**
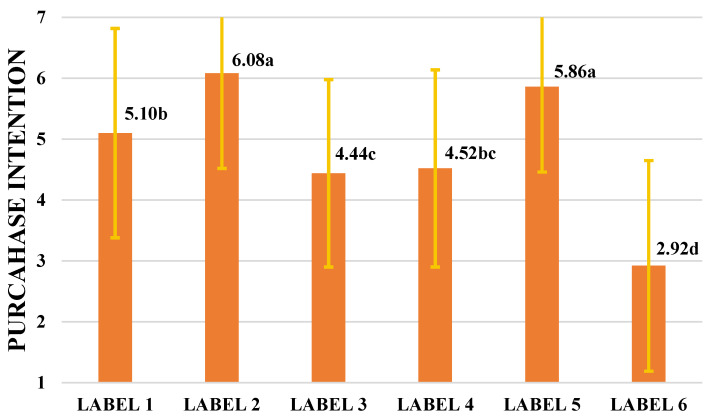
Mean purchase intention scores for the specialty coffee labels; error bars represent standard deviations. Different letters indicate significant differences (ANOVA: F-statistic 63.01, *p*-value 2.4208 × 10^−51^, Tukey’s test: *p* < 0.05). Seven-point structured scale: 1 = “Certainly would not buy” and 7 = “Certainly would buy”.

Figure 4 presents the mean purchase intention (PI) scores for the evaluated labels. Consumers reported higher PI for Labels 2 and 5, which did not differ statistically from each other (*p* > 0.05); both means were close to 6 on the PI scale, corresponding to the “probably would buy” category. Label 1 showed a mean of 5.10, with no significant difference (*p* > 0.05) from Label 4 (mean = 4.52), indicating a PI in the “might buy” range. Likewise, Label 3 (mean = 4.44) did not differ significantly from Label 4 (*p* > 0.05), placing it between “not sure if would buy” and “might buy.” Finally, Label 6 obtained the lowest mean PI and differed statistically from all other labels.

**Figure 5:**
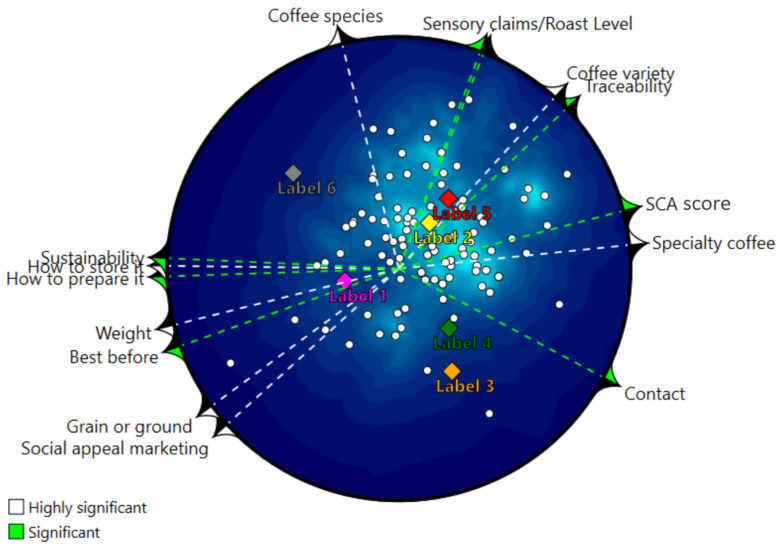
Landscape Segmentation Analysis (LSA) map of purchase intention for the six specialty coffee labels. White dots represent individual consumers’ ideal points (*n* = 105), and colored diamonds indicate the positions of the six labels in the two-dimensional unfolding space. Vectors around the perimeter correspond to informational attributes; arrows indicate the direction in which a higher Normalized Fixation Ratio (NFR) for a given attribute is associated with higher purchase intention. White arrows denote highly significant correlations (*p* ≤ 0.010), and green arrows indicate significant correlations (0.010 < *p* ≤ 0.050).

Figure 5 displays the Landscape Segmentation Analysis (LSA), mapping coffee labels in a two-dimensional space derived from an unfolding solution of purchase-intention scores. The inner white points represent individual consumer ideal points with respect to purchase intention, while the colored diamonds denote the six labels. Around the perimeter, “drivers of purchase intention” are shown; arrows indicate the direction in which a higher Normalized Fixation Ratio (NFR) for a given attribute is associated with higher purchase intention for the evaluated labels. White arrows mark correlations with *p*-value ≤ 0.010, and green arrows indicate 0.010 < *p*-value ≤ 0.050.

Significant attributes (*p*-value ≤ 0.050) showed high correlations between NFR and the LSA preference surface (e.g., r = 0.92 for sensory claims, r = 0.85 for traceability, and r = 0.82 for SCA score; overall range ≈ 0.82–0.97)

## 4. Discussion

The heatmap analyses revealed consistent patterns of consumer visual attention to specialty coffee labels, underscoring the role of strategic design in visual communication. The spatial arrangement of information on labels significantly shapes how consumers process visual stimuli, delineating zones of higher and lower fixation. Such distribution can directly affect product perception and purchase decisions, in line with prior work on visual attention hierarchies [26].

Overall, central regions, on both the front and back panels, showed the longest fixation times. On the front, elements positioned in the upper-central and mid-central areas (e.g., logos, brand names, and descriptors such as “specialty coffee”) captured the bulk of attention. This pattern corroborates [21], who report a tendency for viewers to concentrate on central locations that typically combine higher contrast with greater informational relevance. In addition, the lower-central region(where net weight is often placed) also attracted meaningful fixations, reinforcing the recommendation that core product information be positioned in visually accessible locations [27].

Conversely, the upper lateral corners and lower lateral edges, on both front and back panels, received comparatively little attention. Elements frequently located there (e.g., supplementary seals, opening instructions, or administrative details) elicited few fixations. This pattern suggests that, while such content can be accommodated in peripheral areas, these regions are less effective for communicating key product attributes. The observed distribution aligns with Atalay et al. [27], who emphasize the advantages of placing critical information in central and upper zones to maximize consumer impact.

On the back panels, the upper-central region (often containing narrative descriptions about product history or production sustainability)received substantial attention. These texts appear to leverage both emotional and informational appeal to attract gaze. The central-lower area also stood out, drawing attention to technical attributes such as lot/batch number, best-before date, and preparation instructions.

Together, these findings indicate that central regions play a pivotal role in consumer communication, whereas peripheral areas (less frequently observed)are better suited for lower-priority information. This visual-attention pattern provides clear guidance for optimizing specialty coffee label design: strategically allocate high-value content to central zones to maximize impact at the point of choice.

Moreover, labels featuring longer back-panel narratives showed heightened fixation on the first two opening sentences. This suggests that to optimize comprehension and retention, the most relevant information should be front-loaded at the beginning of the narrative, ensuring it is processed before consumer attention disperses.

The analysis of the Normalized Fixation Ratio (NFR) (Figure 3) reveals consistent patterns of visual behavior across labels, in line with prior research on label attention [6,7]. Overall, some AOIs conveying technical/mandatory information, particularly coffee species, grain or ground, and best before, received intermediate attention when positioned in central or otherwise salient regions. In contrast, other mandatory elements, such as weight and contact, which were usually placed in peripheral areas, consistently ranked among the least attended attributes. These elements carry practical relevance by communicating essential product characteristics, but their ability to attract gaze appears to depend strongly on their graphic placement, as recommended by Bojko [28]. This pattern was especially evident for Label 1, which lacked sensory-quality cues (e.g., sensory claim, SCA score) and origin information (traceability); in this case, mandatory statements attracted comparatively greater attention.

When present, sensory-quality cues emerged as the most visually engaging AOIs. For example, the SCA score reached 5.8 NFR on Label 6, and the sensory claim attained 5.5 NFR on Label 2 (Figure 3). Their salience likely reflects both informational and affective appeal—these cues signal quality, shape expectations, and support decision-making—consistent with Teixeira et al. [15], who highlight the role of sensory descriptors in directing attention and forming expectations. Beyond sensory cues, origin-related information (traceability) (such as farm, region, and altitude) also elicited strong engagement, helping to establish a narrative connection and underscore product distinctiveness. Traceability recorded the highest NFR values on Labels 2 and 5 (5.5 and 3.4, respectively).

Notably, the prominence of these origin cues was not confined to visual attention alone. Crucially, the Landscape Segmentation Analysis (Figure 5) corroborates these patterns behaviorally. A dense cluster of consumer points in the upper-right quadrant—where Labels 2 and 5 are located, aligns with vectors for sensory claims, roast level, coffee variety, traceability, SCA score, and specialty coffee, indicating that longer gaze to these attributes (higher NFR) co-occurs with higher purchase intention. This aligns with Dodd et al. [2] and Teixeira et al. [15], who emphasize the value of origin and sensory quality narratives as effective differentiation strategies in the specialty coffee market.

By contrast, attributes such as weight, best before, grain or ground, social-appeal marketing, and sustainability project toward regions with lower consumer-point density, suggesting weaker or even negative associations with purchase intention. Their associations with purchase intention remain positive and statistically significant (correlations between NFR and the preference surface in the range r ≈ 0.87–0.97; *p* ≤ 0.05), but the directions of the vectors indicate that increased attention to these cues tends to benefit a smaller subset of consumers compared with core sensory and traceability cues.

Technical/mandatory cues appear to function as low-variance information: because nearly all labels display similar values (e.g., net weight, a standard best-before format, grain or ground), these cues carry limited discriminatory power at choice. Moreover, recent studies indicate that environmental claims often underperform relative to more concrete product-quality cues, particularly among less engaged consumers [29]. These results indicate that while mandatory information secures reliable baseline attention, sensory and origin claims are the principal drivers of gaze when available and, critically, are associated with stronger purchase predisposition.

However, it is important to note that the mere presence of information on a label is not sufficient to ensure higher purchase intention. For example, when comparing Label 6 with Labels 5 and 2 in Figure 1, despite sharing many of the same informational elements, Label 6 achieved the lowest purchase intention (2.92) and differed statistically from all other labels (Figure 4). This finding suggests that factors beyond informational content—particularly visual design—also shape purchase decisions. Plassmann et al. [18] showed that consumers base their choices not only on product attributes but also on how these attributes are presented. This relates to visual hierarchy—packaging appearance, color palette, graphic layout, and information formatting can influence perceived quality and willingness to buy even when the technical content remains unchanged [30,31].

Accordingly, our results not only validate the importance of origin and product attributes in shaping consumer perception but also underscore the need for design strategies that optimize label communication. Given that most consumer choices occur rapidly and with limited deliberation [14], positioning key elements strategically in visually accessible regions can substantially enhance product attractiveness and conversion to purchase.

## 5. Conclusions

This study combined eye tracking and purchase intention measures to elucidate how consumers visually process specialty coffee labels and how this processing relates to their purchase intention. Across six commercially available labels, central regions of the front and back panels consistently emerged as attentional “hot zones”, particularly when occupied by brand identifiers and quality-signaling content. Normalized Fixation Ratio (NFR) results showed that, when available, sensory claims, SCA score, roast level, coffee variety, traceability, and the “specialty coffee” designation attracted visual attention, whereas weight, best before, grain or ground, and contact information tended to receive comparatively little attention. For longer textual claims, fixations concentrated mainly on the first two sentences, suggesting that extended narratives are only partially processed. These patterns indicate that consumers prioritize information that conveys quality and distinctiveness over more technical or administrative cues.

By linking NFR values to purchase intention via Landscape Segmentation Analysis, the study demonstrated that some attributes attracting the most attention were also those most strongly associated with higher purchase intention. In particular, traceability information and quality cues such as SCA score and sensory claims were positively related to purchase intention. To maximize PI, label designers should, therefore, ensure that information is not only present but made graphically salient and positioned in central visual “hot zones”, with social-appeal and sustainability messages playing a supporting rather than leading role in the overall composition. These principles may extend to other premium food and beverage categories where origin and sensory differentiation are central to value creation.

Importantly, however, high attention did not always translate into a strong positive impact on purchase intention. Social-appeal marketing and sustainability messages sometimes received substantial visual attention (e.g., on Labels 1 and 5), yet their LSA vectors showed comparatively weaker gradients toward regions with high consumer-point density. This suggests that, although these cues attract gaze, they may be perceived as less diagnostic for product quality than sensory and origin information, and in some cases may even elicit skepticism about the credibility of the claims. Consequently, such information is likely to play a supporting rather than a leading role in the overall label composition. The results, therefore, provide concrete guidance for specialty coffee producers, designers, and marketers on how to prioritize and stage different informational elements.

Despite its significant contributions, some limitations should be acknowledged. The use of biometric sensors, such as eye tracking, electroencephalography (EEG), facial-expression analysis, or autonomic indices (e.g., heart rate and skin conductance), represents a passive method, capturing consumers’ visual attention without requiring an explicit response. However, future research could achieve a more comprehensive understanding of consumer perception by combining this approach with active methods, in which participants explicitly express their perceptions and judgments (e.g., text highlighting, check-all-that-apply (CATA), and free-listing)allowing researchers to evaluate not only the information presented on the labels, but also how it is displayed, including the overall design such as color, and pattern of label elements. Such multimethod designs would offer a richer picture of how specialty coffee labels create value at both conscious and non-conscious levels.

## Data Availability

Data are available from the corresponding authors upon reasonable request.

## Abbreviations

The following abbreviations are used in this manuscript:

AOI	Area of Interest
NFR	Normalized Fixation Ratio
SCA	Specialty Coffee Association
LSA	Landscape Segmentation Analysis
PI	Purchase Intention
CA	Correspondence Analysis
